# The Clinical Effectiveness of Cognitive Behavioral Therapy for Patients with Insomnia and Depression: A Systematic Review and Meta-Analysis

**DOI:** 10.1155/2020/8071821

**Published:** 2020-07-15

**Authors:** Guiyu Feng, Mei Han, Xun Li, Le Geng, Yingchun Miao

**Affiliations:** ^1^Dongzhimen Hospital, Beijing University of Chinese Medicine, Beijng 100700, China; ^2^School of Traditional Chinese Medicine, Beijing University of Chinese Medicine, Beijing 100029, China

## Abstract

**Background:**

Insomnia and depression often co-occurr. However, there is lack of effective treatment for such comorbidity. CBT-I has been recommended as the first-line treatment for insomnia; whether it is also effective for comorbidity of insomnia and depression is still unknown. Therefore, we conducted this meta-analysis of randomized controlled trials to assess the clinical effectiveness and safety of CBT-I for insomnia comorbid with depression. *Data Sources.* Seven electronic databases, including China National Knowledge Infrastructure (CNKI), Wanfang Database, China Science Technology Journal Database, SinoMed Database, PubMed, the Cochrane Library, and EMBASE, as well as grey literature, were searched from the beginning of each database to July 1, 2019. *Study Eligibility Criteria.* Randomized controlled trials that compared CBT-I to no treatment or hypnotics (zopiclone, estazolam, and benzodiazepine agonist) for insomnia comorbid with depression and reported both insomnia scales and depression scales. *Study Assessment and Synthesis Methods.* Cochrane Reviewer's Handbook was used for evaluating the risk of bias of included studies. Review Manager 5.3 software was used for meta-analysis. Online GRADEpro was used to assess the quality of evidence.

**Results:**

The pooled data showed that CBT-I was superior to no treatment for insomnia, while it was unsure whether CBT-I was better than no treatment for depression. And the effectiveness of CBT-I was comparable to hypnotics for both insomnia and depression. CBT-I was likely to be safe due to its noninvasive nature. The methodological quality varied across these trials. The evidence quality varied from moderate to very low, and the recommendation level was low.

**Conclusions:**

Currently, findings support that CBT-I seems to be effective and safe for insomnia comorbid with depression to improve the insomnia condition, while it is unsure whether CBT-I could improve depression condition. More rigorous trials are needed to confirm our findings.

## 1. Introduction

Insomnia is a kind of sleep disorder, and people with insomnia are unsatisfied with their sleep time and sleep quality. Insomnia patients have difficulty initiating, maintaining sleep or returning to sleep [1]. Several studies have shown that 6% to 10% of adults suffered from insomnia [2–4], and 10–15% of insomnia patients tended to develop into chronic course [5]. Insomnia increases the risk of many health problems including suicidal ideation and behavior [6], cardiovascular diseases [7], depressive disorder, arterial hypertension, myocardial infarction [8], chronic heart failure [7, 9], type 2 diabetes [10], and cognitive impairment [11], which would place a heavy burden on society and individuals. Treatments for insomnia included cognitive behavioral therapy, pharmacologic therapy, and complementary and alternative therapy. Pharmacologic therapy for insomnia includes benzodiazepines (triazolam, estazolam, temazepam, flurazepam, and quazepam), nonbenzodiazepine hypnotics (zaleplon, zolpidem, and eszopiclone). Complementary and alternative therapy for insomnia includes acupuncture and Chinese herbal medicine. Moderate-quality evidence showed that CBT-I improved sleep outcomes in the general population, including reduced sleep onset latency and wake after sleep onset, and improved sleep efficiency and sleep quality. And any harm associated with CBT-I is likely to be mild, so CBT-I was recommended as the first-line treatment by the ACP Clinical Guidelines Committee [12, 13].

In fact, comorbid insomnia is commonly seen in clinical practice. And insomnia always combines with depression. Symptoms of depression include depressive mood, decreased interest, lack of motivation, and fatigue [14]. Insomnia and depression are often influenced by each other, and this mutual influence may increase the risk of suicide. A recent study suggests that sleep problems are associated with severe depression, suicidality, and worse outcomes for treatment of depression [15]. And another study shows that early changes in insomnia characteristics may predict long-term depression outcomes [16]. No therapy has been proven to be effective and safe for this comorbidity at present. Sequential approach and concomitant treatment for such comorbidity are both in their preliminary stages; well-designed randomized controlled trials with long-term follow-up are needed to evaluate the effectiveness and safety of these treatments.

Cognitive behavioral therapy is commonly used in clinical practice, which includes cognitive behavioral therapy for insomnia, cognitive behavioral therapy for depression [17], cognitive behavioral therapy for psychosis [18], brief behavioral treatment for insomnia [19], and cognitive behavioral therapy for Parkinson [20]. Among these therapies, cognitive behavioral therapy for insomnia (CBT-I), developed by A.T. Beck in the 1960s, is a branch of CBT and is recommended as first-line treatment for insomnia by the ACP Clinical Guidelines Committee in 2016. The components of CBT-I include cognitive therapy to replace wrong beliefs of sleep; stimulate control to prevent patients from associating with other stimulating activities; sleep restriction to limit time in bed to match perceived sleep duration; sleep hygiene to change habits and physiologic factors and improve sleep; and relaxation to focus on relaxation techniques, such as guided imagery and progressive muscle relaxation (Table 1) [21].Various delivery methods of CBT-I are available, including in-person individual or group therapy, telephone- or Web-based modules, and self-help books. The course of CBT-I varied from 4 sessions to 12 sessions.

We conducted this meta-analysis of randomized controlled trials to assess the clinical effectiveness and safety of CBT-I for insomnia comorbid with depression, compared with no treatment or hypnotics and measured by insomnia outcome measurements, depression outcome measurements, and safety index.

## 2. Methods

### 2.1. Protocol and Registration

We made this systematic review and meta-analysis according to the Preferred Reporting Items for Systematic Reviews and Meta-Analyses (PRISMA). We registered this review in PROSPERO (CRD42019145065), http://www.crd.york.ac.uk/PROSPERO/.

### 2.2. Inclusion and Exclusion Criteria

#### 2.2.1. Inclusion Criteria

1. Participants (aged ≥18 years, regardless of gender and education) met the diagnostic criteria for insomnia and depression, which referred to DSM-5 and CCMD-3.
2. Intervention of the experimental group was only CBT-I.
3. Comparison (control group) included no treatment and hypnotic drugs (zopiclone, estazolam, and benzodiazepine agonist). No treatment referred to no treatment for insomnia and depression during observation period. Waitlist control had no treatment for insomnia and depression during observation period, so we also took waitlist control as no treatment. We did not take sleep hygiene education alone as the control for CBT-I contained sleep hygiene education.
4. Outcome measurements included insomnia scale, depression scale, and adverse events. Insomnia scales included ISI and PSQI. Depression scale included HAMD, HADS-D, BDI, and SDS (primary outcomes) and SCL-90, CES-D, and QIDS-CR16 (secondary outcomes). Adverse events included all adverse events of CBT-I and hypnotics mentioned by these RCTs included in our review.
5. Study included randomized controlled trials (RCTs) which focused on Chinese language and English language.

#### 2.2.2. Exclusion Criteria

1. Patients could not be diagnosed with insomnia and depression
2. Intervention was not only CBT-I, such as CBT-I plus other therapies
3. Control group was neither no treatment nor hypnotics
4. Outcomes reported incompletely, such as only reported insomnia scales or only reported depression scales
5. Trials did not mention RCT or the word “random”
6. Duplication of the study
7. Study was mechanism, case report, review, or meta-analysis
8. Study language was neither Chinese nor English

### 2.3. Literature Search and Data Extraction

Guiyu Feng and Le Geng independently searched PubMed, the Cochrane Library, EMBASE, SinoMed Database, China National Knowledge Infrastructure (CNKI), Wanfang Database, and China Science Technology Journal Database from the beginning of each database to July 1, 2019 (CNKI: 1915–2019, Wanfang: 1900–2019, VIP: 1989–2019, SinoMed: 1860–2019, PubMed: 1966–2019, EMBASE: 1947–2019, and Cochrane: 1966–2019). The details of search strategy of PubMed were shown in Appendix. Grey literature was also searched to identify potential studies which met the inclusion and exclusion criteria. They focused on languages of English and Chinese. They also searched relevant RCTs from existing systematic review and meta-analysis in the reference list. They downloaded search results for evaluation. They also contacted authors whose RCTs lacked any relevant information. And they also independently screened the literatures and found out suitable RCTs at the same time, according to inclusion and exclusion criteria. After literature selection, they separately extracted data from these suitable RCTs at the same time. Extracted data included author's name, year of publication, sample size, age, intervention, underlying disease, control group, duration of treatment, time point of assessment, and outcomes. If any disagreement happened, they would resolve it in consultation with more experienced author Yingchun Miao. Yingchun Miao conducted the search, Mei Han evaluated the abstract, and Xun Li evaluated the rest of the paper.

### 2.4. Outcome Measurements

The outcome measurements of this systematic review and meta-analysis included insomnia outcome measurements, depression outcome measurements, and adverse events. More details of outcome measurements are shown in Table 2.

### 2.5. Risk of Bias

Guiyu Feng and Le Geng independently evaluated the risk of bias through the Cochrane Handbook for Systematic Reviews of Interventions [22] to evaluate the methodological quality of these included literatures and they performed it via Review Manager 5.3 at the same time. For all evaluation items, the quality of each trial was evaluated using “Yes” (low risk of bias), “No” (high risk of bias), or “Unclear” (unclear risk of bias). Evaluation items included random sequence generation, allocation concealment, blinding of participants and personnel, blinding of outcome assessment, incomplete outcome data, selective reporting, and other biases. If any disagreement happened, they would resolve it in consultation with more experienced author Yingchun Miao.

### 2.6. Data Synthesis

We used Cochrane collaboration software RevMan (5.3) to pool outcome data. We calculated the risk ratio (RR) with 95% confidence interval (95% CI) for dichotomous variables and mean difference (MD) with 95% CI for continuous outcomes. Outcomes of insomnia and depression were all continuous variables, and we used mean difference (MD) and its 95% confidence interval (CI) to represent them. Heterogeneity was evaluated by the magnitude of Tau^2^ and I^2^ statistic. A fixed effect model was performed with minor heterogeneity when the I^2^ value was below 50%. A random effect model was used with major heterogeneity when the I^2^ value was above 50%. For patient population with underlying diseases, these underlying diseases were different, such as posttraumatic stress disorder (PTSD), ischemic stroke, hypertension, and nonmetastatic cancer. Heterogeneity was major; so, we also performed the random effect model. We would do subgroup analysis and sensitive analysis if the characteristics of data were allowed. If the number of suitable RCTs was more than 10, we would make an inverted funnel plot to assess the impact of publication bias.

### 2.7. Evaluating the Quality of Evidence

Guiyu Feng and Le Geng independently used the online GRADEpro to assess the quality of evidence (https://gdt.gradepro.org/app/#) at the same time.

## 3. Results

### 3.1. Literature Screening and Its Flow Diagram

**Figure 1:**
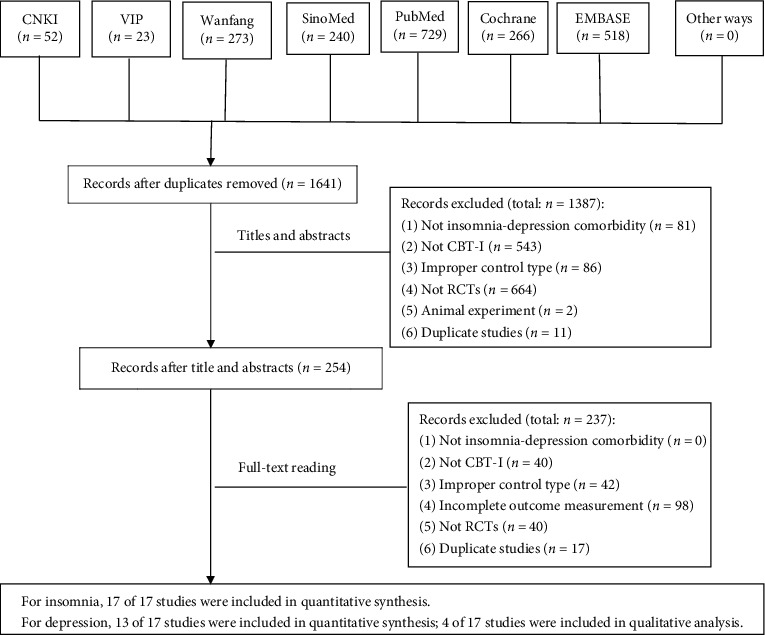
Study flow diagram.

We searched 2,101 RCTs according to the search strategy. After duplicated RCTs were deleted, there were 1,641 RCTs left. When we screened the titles and abstracts, 1,387 RCTs which did not meet search criteria were deleted. We then screened the full texts of the remaining 254 RCTs and found 237 RCTs which did not meet search criteria. In the end, 17 RCTs were included in this review. More details of literature screening are shown in Figure 1.

### 3.2. Characteristics of Included Literature Studies

We finally included 17 RCTs and 1,756 participants (894 participants in the intervention group and 862 participants in the control group). 13 RCTs [23–35] compared CBT-I with no treatment. Four RCTs [36–39] compared CBT-I with hypnotics (zopiclone and estazolam). One RCT [32] reported two types of CBT-I, and the remaining 16 RCTs [23–31, 33–39] all reported one type of CBT-I arms. The mean frequency of CBT-I was about once a week. Two RCTs [36, 37] used zopiclone 3.75～11.25 mg QN, 1 RCT [38] used estazolam 1 mg QN, and 1 RCT [39] did not mention the dosage of benzodiazepine agonist. The mean duration of treatment was about 8 weeks. The mean assessment time point was about week 8. The underlying diseases differed in this review, including nonmetastatic cancer, ischemic stroke, maintain hemodialysis (MHD), hypertension, poststroke fatigue, hearing impairment, and posttraumatic stress disorder (PTSD). More details are shown in Table 3.

### 3.3. Methodological Quality Evaluation

In order to evaluate the methodological quality of these included literature studies, we used the Cochrane Handbook for Systematic Reviews of Interventions. The overall methodological quality was not good.

For random sequence generation, 15 RCTs [23–35, 37, 39] used the right methods to produce the random sequence, and we assessed them as ‘low' risk. One RCT [36] did not mention how to produce the random sequence, except the word ‘random,' so we assessed them as ‘unclear' risk. 1 RCT [38] used wrong method to produce the random sequence, for it used enrollment order of the facilities, for example, the first enrolled facility was allocated to the CBT-I group while the second to the control group. We assessed it as ‘high' risk.

For allocation concealment, 5 RCTs [23, 30, 31, 34, 35] mentioned the right methods of allocation concealment, and we assessed them as ‘low' risk. 12 RCTs [24–29, 32, 33, 36–39] did not mention how to make allocation concealment, so we evaluated them as ‘unclear' risk.

For blinding of participants and personnel, due to the characteristics of CBT-I, it was hard to blind doctors and patients. So, we assessed all of them as ‘high' risk. For blinding of outcome assessment, 4 RCTs [23, 31, 34, 35] blinded the outcome assessors, and we assessed them as ‘low' risk. 13 RCTs [24–30, 32, 33, 36–39] did not mention blinding of outcome assessment. We did not know whether they blinded outcome assessors, so we evaluated them as ‘unclear' risk.

For incomplete outcome data, 16 RCTs [23–25, 27–39] reported the drop-outs, lost patients, and the reasons, so we assessed them as ‘low' risk. One RCT [26] did not mention incomplete outcome data. We did not know whether there were incomplete outcome data, so we assessed it as ‘unclear' risk.

For selective reporting, all 17 RCTs [23–39] reported the outcomes of insomnia scales and depression scales, and we assessed all of them as ‘low' risk.

For other biases, we focused on whether the baseline was equal between the intervention group and the control group. There was no statistic difference between the intervention group and the control group in all 17 RCTs [23–39]. We assessed all of them as ‘low' risk.

**Figure 2:**
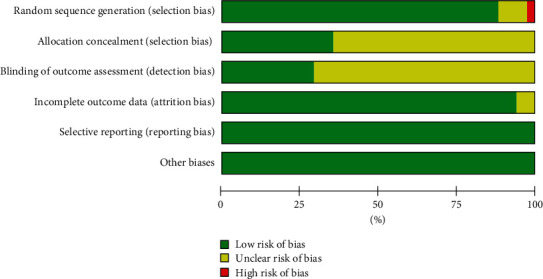
Risk of bias.

Details of methodological quality evaluation are shown in Figure 2.

### 3.4. Results of Meta-Analysis

Based on the presence or absence of underlying diseases, patients were divided into 2 groups including patients with underlying diseases and patients without underlying diseases.

#### 3.4.1. Patients with Underlying Diseases

**Figure 3:**
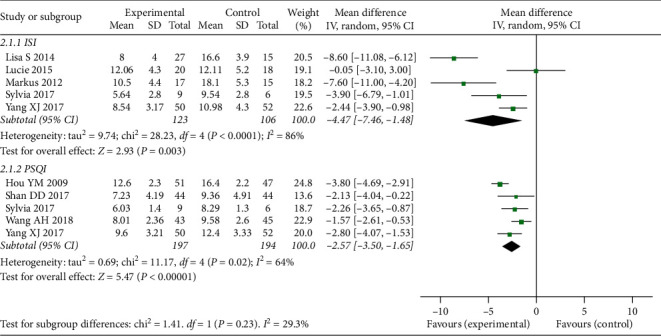
Forest plot of insomnia outcomes (CBT compared to no treatment in patients with underlying diseases).

*(1) Insomnia Outcome Measurements*. In comparison between CBT-I and no treatment, we included 5 RCTs [23, 28–30, 35] (total 229 participants) for ISI and 5 RCTs [25–29] (total 391 participants) for PSQI. The results of meta-analyses showed that CBT-I was more effective than no treatment (MD-4.47, 95% CI [−7.46, −1.48], I^2^ = 86%; MD-2.57, 95% CI [−3.50, −1.65], I^2^ = 64% for ISI scores and PSQI scores, respectively). More details of the meta-analysis are shown in Figure 3.

**Figure 4:**
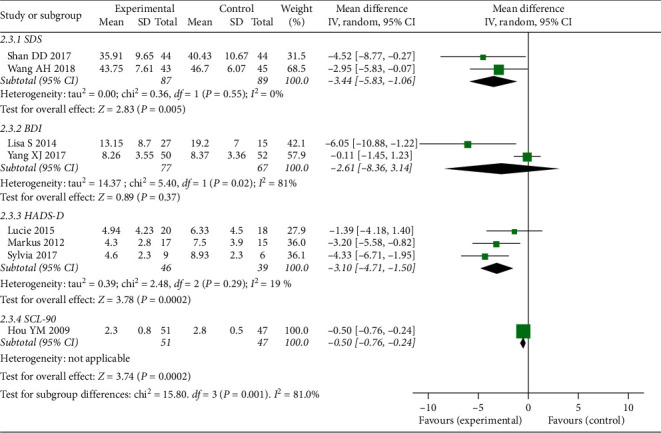
Forest plot of depression outcomes (CBT compared to no treatment in patients with underlying diseases).

*(2) Depression Outcome Measurements*. In comparison between CBT-I and no treatment, we included 2 RCTs [25, 26] (total 176 participants) for SDS scores; 3 RCTs [23, 29, 30] (total 85 participants) for HADS-D scores; 1 RCT [27] (total 98 participants) for SCL-90 scores; and 2 RCTs [28, 35] (total 144 participants) for BDI scores. The results of meta-analyses showed that CBT-I was more effective than no treatment (MD-3.44, 95% CI [−5.83, −1.06], I^2^ = 0%; MD −3.10, 95%CI [−4.71, −1.50], I^2^ = 19% for SDS scores and HADS-D scores, respectively). The result of SCL-90 scores showed that CBT-I was more effective than no treatment (MD-0.50, 95% CI [−0.76, −0.24]). However, the result of meta-analysis in BDI scores showed that there was no significant difference between CBT-I and no treatment with MD-2.61, 95% CI [−8.36, 3.14], I^2^ = 81%. More details of the meta-analysis are shown in Figure 4.

#### 3.4.2. Patients without Underlying Diseases

**Figure 5:**
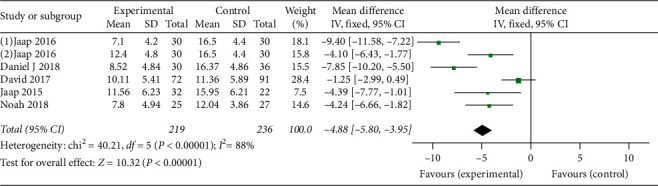
Forest plot of insomnia outcomes (CBT compared to no treatment in patients without underlying diseases).

*(1) Insomnia Outcome Measurements*. In comparison between CBT-I and no treatment, we included 5 RCTs [24, 31–34] (total 455 participants) for ISI. The result of meta-analysis showed that CBT-I was more effective than no treatment with MD-4.88, 95% CI [−5.80, −3.95], I^2^ = 88%. More details of the meta-analysis are shown in Figure 5.

**Figure 6:**
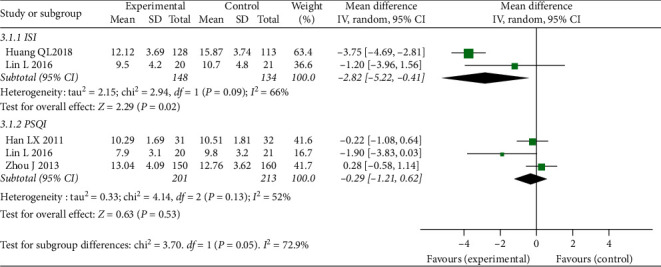
Forest plot of insomnia outcomes (CBT compared to hypnotics in patients without underlying diseases).

In comparison between CBT-I and hypnotics, we included 2 RCTs [37, 39] (total 282 participants) for ISI scores and 3 RCTs [36, 38, 39] (total 414 participants) for PSQI scores. The result of meta-analysis in ISI scores showed that CBT-I was superior to hypnotics with MD-2.82, 95% CI [−5.22, −0.41], I^2^ = 66%. However, the result of meta-analysis in PSQI scores showed that there was no significant difference between CBT-I and hypnotics for insomnia with MD-0.29, 95% CI [−1.21, 0.62], I^2^ = 52. More details are shown in Figure 6.

**Figure 7:**
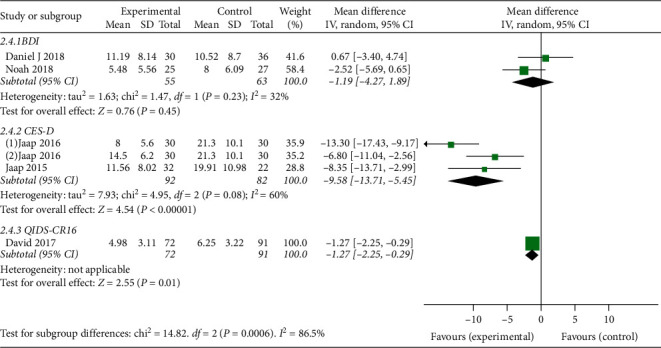
Forest plot of depression outcomes (CBT compared to no treatment in patients without underlying diseases).

*(2) Depression Outcome Measurements*. In comparison between CBT-I and no treatment, we included 2 RCTs [24, 34] (total 118 participants) for BDI; 2 RCTs [32, 33] (total 174 participants) for CES-D, and 1 RCT [31] (total 163 participants) for QIDS-CR16. The result of meta-analysis showed that CBT-I was more effective than no treatment with MD-9.58, 95% CI [−13.71, −5.45], I^2^ = 60% in CES-D. The result in QIDS-CR16 illustrated that CBT-I was more effective than no treatment with MD-1.27, 95% CI [−2.25, −0.29]. However, the result of meta-analysis in BDI indicated that there was no significant difference between CBT-I and no treatment for depression with MD-1.19, 95% CI [−4.27, 1.89], I^2^ = 32%. More details of the meta-analysis are shown in Figure 7.

**Figure 8:**
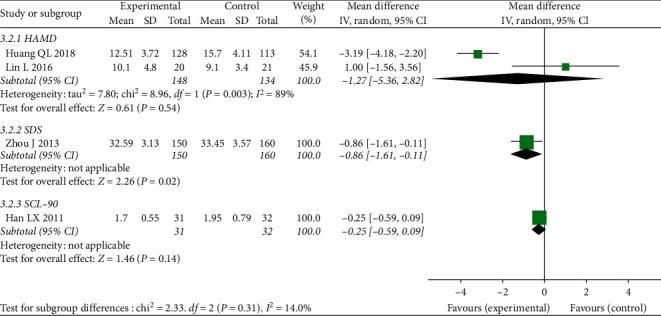
Forest plot of depression outcomes (CBT compared to hypnotics in patients without underlying diseases).

In comparison between CBT-I and hypnotics, we included 2 RCTs [37, 39] (total 282 participants) for HAMD scores; 1 RCT [38] (total 310 participants) for SDS scores; and 1 RCT [36] (63 participants) for SCL-90 scores. The result of meta-analysis in HAMD showed that there was no significant difference between CBT-I and hypnotics with MD-1.27, 95% CI [−5.36, 2.82], I^2^ = 89%. The result of SDS scores showed that CBT-I was more effective than hypnotics with MD-0.86, 95% CI [−1.61, −0.11]. The result of SCL-90 showed that there was no significant difference between CBT-I and hypnotics (zopiclone and estazolam) with MD-0.25, 95% CI [−0.59, 0.09]. More details are shown in Figure 8.

#### 3.4.3. Adverse Event

From these included studies [23–39], only 1 RCT [32], comparing CBT-I with no treatment, reported the item of adverse event, which represented that there was no adverse event which occurred in CBT-I and no treatment.

#### 3.4.4. Publication Bias

For the number of included studies in each of these outcomes was less than 10, we could not make an inverted funnel plot to assess the influence of publication bias of included studies.

### 3.5. Evaluating the Quality of Evidence

The quality of evidence was evaluated via GRADEpro. The quality of evidence in our review varied from moderate to very low. For patients with underlying diseases, the evidence of insomnia outcome measurements was moderate; the evidence of depression outcome measurements varied from low to very low. For patients without underlying diseases, the evidence of insomnia outcome measurements varied from moderate to low; the evidence of depression outcome measurements varied from low to very low. More details are shown in Table 4.

The recommendation level assessed by the GRADE system is based on the factors including the advantages of CBT-I, the evidence quality in our review, the preferences of patients, and the cost of CBT-I. The evidence quality varied from moderate to very low in our review; CBT-I is receptible for the patients due to its noninvasive characteristics. And the cost of CBT-I is not so frightfully expensive. So, the recommendation level of the evidence in our review is low.

## 4. Discussion

### 4.1. Summary of Results

Insomnia scale, depression scale, and adverse event were included in our review to assess the effectiveness and safety of CBT-I. Based on the presence or absence of underlying diseases, we divided patients into 2 groups including patients with underlying diseases and patients without underlying diseases. Our results showed that CBT-I was an effective therapy for insomnia, while CBT-I was not an effective therapy for depression in patients suffering from insomnia and depression. And CBT-I was as effective as hypnotics (zopiclone, estazolam, or benzodiazepine agonist) for insomnia, and both CBT-I and hypnotics were not effective for depression. CBT-I was likely to be a safe therapy due to its noninvasive characteristics. The methodological quality was not good enough. The evidence quality varied from moderate to very low, and the recommendation level based on the evidence was low.

### 4.2. Comparison with the Previous Study

A number of reviews mentioned the effectiveness of CBT for insomnia or depression, respectively. For example, one review [40] assessed the effectiveness of self-help CBT-I for insomnia by comparing it with waiting list control, routine care, or no treatment, and the result showed that self-help CBT-I was significantly more effective than waiting list control, routine care, or no treatment; another review [41] assessed the effectiveness of online cognitive behavioral therapy (OCBT) for postpartum depressive symptomatology by comparing it with waiting list or treatment as usual. And the result identified a moderate significant size effect (*d* = −0.54, 95% CI [−0.716; −0.423]) of OCBT in reducing postpartum depression. However, few reviews focused on CBT for insomnia comorbidity with depression; therefore, our review assessed the effectiveness of CBT-I for insomnia comorbidity with depression by comparing it with no treatment or hypnotics. And the results of our review showed CBT-I was an effective therapy for patients with insomnia comorbid with depression to some degree. And the clinical effectiveness of CBT-I and hypnotics was familiar with no significant difference between CBT-I and hypnotics.

### 4.3. Strengths and Limitations

As we mentioned above, a number of reviews focused on the effectiveness of CBT-I for insomnia alone or depression alone. Considering insomnia often co-occurred with depression clinically, there were few guidelines or reviews focused on CBT-I for insomnia comorbidity with depression, so our review assessed the effectiveness of CBT-I for insomnia comorbidity with depression to provide evidence for clinical practice.

Limitations at review level:the quality of evidence varied from moderate to very low; the number of studies in each outcome was less than 10, we could not make publication bias. Limitations at study level: the number of qualified RCTs was insufficient, and the sample size of included studies was small. Limitation at outcome level: only one RCT mentioned the item of adverse event and showed that no harm occurred in both CBT-I and no treatment; more evidence is needed to confirm the safety of CBT-I.

### 4.4. Implications for Clinical Practice

The evidence of our review supported that CBT-I was effective for insomnia in patients suffering from insomnia and depression. And the effectiveness was comparable to hypnotics. CBT-I was likely to be safe due to its noninvasive nature. The findings suggest that CBT-I confers beneficial effects. Because of the low recommendation level of evidence, practitioners could recommend this therapy to patients and finally make a decision based on the evidence, the experience of doctors, and the preferences of patients.

### 4.5. Implications for Future Research

(1) Future studies should be conducted according to the Consolidated Standards of Reporting Trials (CONSORT) statement, which is essential to control the risk of bias. For example, considering the characteristics of CBT-I, it is hard to blind the doctors and patients, but we could blind outcome assessors. We could also pay more attention to allocation concealment to improve methodological quality of future studies. (2) Researchers should report every detail of studies according to the CONSORT statement, for example, adverse events in CBT-I should be comprehensively reported, although CBT-I appeared to be safe.

## 5. Conclusion

CBT-I may be an effective therapy for insomnia, and the effectiveness of CBT-I is comparable to hypnotics, while CBT-I is not an effective therapy for depression, which is the same to hypnotics in patients suffering from insomnia and depression. And CBT-I is likely to be safe. However, the quality and quantity of eligible RCTs are not good enough. And the evidence quality varied from moderate to very low, and the recommendation level of evidence was low (↑?/2). Therefore, more well-designed trials are needed to confirm our findings.

## Figures and Tables

**Table 1:** Components of CBT-I.

Component	Description
Cognitive therapy	Cognitive therapy aims to identify and replace dysfunctional beliefs and attitudes about sleep. These dysfunctional beliefs include unrealistic expectations of sleep, such as overestimating the consequences of poor sleep.
Stimulus control	Stimulus control aims at avoiding patients to associate bed with other stimulating activities such as avoiding nonsleep activities in the bedroom; going to bed only when sleepy; and leaving the bedroom when unable to sleep for15−20 min, returning to bed only when sleepy
Sleep restriction	Sleep restriction aims to limit time in bed to match perceived sleep duration in order to increase sleep drive and reduce time awake in bed. Time allowed in bed is initially restricted to the average time perceived as sleep per night and then adjusted to ensure that sleep efficiency remains >85%.
Sleep hygiene	Sleep hygiene relates to environmental factors, physiologic factors, and habits that improve sleep, such as regular sleep scheduling, avoiding long daytime naps, and limiting alcohol, caffeine, and nicotine intake especially before bed.
Relaxation	Any relaxation technique that the patient finds effective can be used to limit cognitive arousal and reduce muscular tension to improve sleep. Specific relaxation techniques include meditation, mindfulness, progressive muscle relaxation, guided imagery, and breathing techniques.

**Table 2:** Details of outcome measurements.

Insomnia outcome measurements		Depression outcome measurements	
Primary outcomes	Secondary outcomes	Primary outcomes	Secondary outcomes
ISI, PSQI	None	HAMD, HADS-D	SCL-90, CES-D
		BDI, SDS	QIDS-CR16

*Note.* ISI: Insomnia Severity Index; PSQI: Pittsburgh Sleep Quality Index; HAMD: Hamilton Depression Scale; HADS-D: Anxiety and Depression Scale-Depression; BDI: Beck Depression Inventory; SDS: Self-Rating Depression Scale; SCL-90: Symptom Checklist 90; CES-D: Centre of Epidemiological Studies Depression Scale (CES-D); and QIDS-CR16: Quick Inventory of Depressive Symptomatology—Clinician Rating.

**Table 3:** Characteristics of included RCTs.

Study ID	Participants (M/F)	Age (years)	Intervention	Underlying disease	Medicine used for underlying disease	Control	Duration of treatment (I/C)	Time point of assessment	Outcomes
Casault et al. [23]	I: 1/19 C: 2/16	I: 56.9 ± 10.8 C: 57.0 ± 9.4	CBT-I (self-administered) weekly	Nonmetastatic cancer	None	No treatment	6 weeks/6 weeks	Week 6	ISI, HADS-D
Daniel et al. [24]	I: 62/13 C: 62/14	I: 32.21 ± 7.18 C: 32.67 ± 7.97	CBT-I, weekly	None	None	No treatment	6 weeks/6 weeks	Week 6	ISI, BDI
Shan [25]	I: 26/18 C: 24/20	I: 54.22 ± 8.39 C: 54.23 ± 8.42	CBT-I, weekly	Ischemic stroke	Aspirin; simvastatin	No treatment	4 weeks/4 weeks	Week 4	PSQI,SDS
Wang et al. [26]	I: 18/25 C: 18/27	I: 57.19 ± 8.51 C: 56.73 ± 8.96	CBT-I, weekly	Various types of cancer	Chemotherapy drugs	No treatment	4 weeks/4 weeks	Week 4	PSQI,SDS
Hou et al. [27]	I: 20/31 C: 22/25	I: 54.5 ± 13.8 C: 52.4 ± 14.5	CBT-I, weekly	Maintain hemodialysis (MHD)	None	No treatment	12 weeks/12 weeks	Week 12	PSQI, SCL-90
Yang et al. [28]	I: 20/30 C: 19/34	I: 56.54 ± 9.97 C: 56.73 ± 11.27	CBT-I (remote-interactive), more than once a week	Hypertension	Antihypertensive drugs	No treatment	8 weeks/8 weeks	Week 8	ISI,PSQI, BDI
Sylvia et al. [29]	I: 6/3 C: 5/1	I: 47.22 ± 15.21 C: 51.17 ± 10.65	CBT-I (individual), weekly	Poststroke fatigue	Medicine (specific drugs not available)	No treatment (waitlist control)	8 weeks/8 weeks	Week 8	ISI,PSQI,HADS-D
Markus et al. [30]	I: 7/10 C: 5/10	I: 57.8 ± 6.6 C: 53.6 ± 10.4	CBT-I, weekly	Hearing impairment	Not mentioned	No treatment (waitlist control)	7 weeks/7 weeks	Week 8	ISI, HADS-D
David et al. [31]	I: 20/82 C: 20/82	I: 44.66 ± 11.65 C: 43.75 ± 11.84	CBT-I (web-based), weekly	None	None	No treatment (waitlist control)	6 weeks/6 weeks	Week 7	ISI,QIDS-CR16
Lancee et al. [32]	I: 6/30 C: 7/20	I: 47.47 ± 14.37 C: 49.98 ± 13.71	CBT-I (online), weekly	None	None	No treatment (waitlist control)	12 weeks/12 weeks	Week 12	ISI, CES-D, adverse event
(1) Lancee et al. [33]	I: 4/26 C: 5/25	I: 41.2 ± 14.1 C: 45.1 ± 13.7	CBT-I (online), weekly	None	None	No treatment (waitlist control)	12 weeks/12 weeks	Week 12	ISI, CES-D
(2) Lancee et al. [33]	I: 8/22 C: 5/25	I: 38.5 ± 13.1 C: 45.1 ± 13.7	CBT-I (individual, face to face), weekly	None	None	No treatment (waitlist control)	12 weeks/12 weeks	Week 12	ISI, CES-D
Lorenz et al. [34]	I: 8/21 C: 9/18	I: 41.72 ± 17.31 C: 44.04 ± 20.05	CBT-I (web-based), weekly	None	None	No treatment (waitlist control)	6 weeks/6 weeks	Week 6	ISI,BDI
Talbot et al.[35]	I: 7/22 C :7/9	I: 37.1 ± 10.4 C: 37.3 ± 11.0	CBT-I (individual), weekly	Posttraumatic stress disorder (PTSD)	Medicine (specific drugs not available)	No treatment (waitlist control)	8 weeks/8 weeks	Week 8	ISI,BDI
Han and Liu [36]	I: 14/17 C: 14/18	I: 37 ± 14 C: 35 ± 14	CBT-I, weekly	None	None	Zopiclone 3.75～11.25 mg QN	8 weeks/8 weeks	Week 8	PSQI,SCL-90
Huang et al. [37]	I: 35/93 C: 28/85	I: 46.78 ± 13.75 C: 45.49 ± 12.83	CBT-I (group), weekly	None	None	Zopiclone 3.75～7.5 mg QN	8 weeks/8 weeks	Week 8	ISI,HAMD
Zhou et al. [38]	150/160	None	CBT-I, weekly	None	None	Estazolam 1 mg QN	6 weeks/6 weeks	Week 6	PSQI,SDS
Lin et al. [39]	I: 4/16 C: 4/17	I: 46.5 ± 12.5 C: 45.5 ± 12.4	CBT-I (remote-interactive), weekly	None	None	Benzodiazepine agonist	8 weeks/8 weeks	Week 8	ISI,PSQI, HAMD

*Note.* M: male; F: female; I: intervention; C: control; QN: once a night; MHD: maintain hemodialysis disorder; PTSD: posttraumatic stress; ISI: Insomnia Severity Index; PSQI: Pittsburgh Sleep Quality Index; HAMD: Hamilton Depression Scale; HADS-D: Anxiety and Depression Scale-Depression; BDI: Beck Depression Inventory; SDS: Self-Rating Depression Scale; SCL-90: Symptom Checklist 90; CES-D: Centre of Epidemiological Studies Depression Scale; and QIDS-CR16: Quick Inventory of Depressive Symptomatology—Clinician Rating.

**Table 4:** Summary of findings table.

	Participants (RCTs)	Quality of evidence	Anticipated absolute effects
			Risk difference with intervention (95% CI)
Patients with underlying diseases			
Insomnia outcome measurements			
CBT-I vs. no treatment			
ISI	229 (5)	⊕⊕⊕⃝moderate	MD 4.47 lower (7.46 lower to 1.48 lower)
PSQI	391 (5)	⊕⊕⊕⃝moderate	MD 2.57 lower (3.5 lower to 1.65 lower)
CBT-I vs. hypnotics	None	None	None
Depression outcome measurements			
CBT-I vs. no treatment			
SDS	176 (2)	⊕⊕⃝⃝low	MD 3.44 lower (5.83 lower to 1.06 lower)
BDI	144 (2)	⊕⃝⃝⃝very low	MD 2.61 lower (8.36 lower to 3.14 higher)
HADS-D	85 (3)	⊕⊕⃝⃝low	MD 3.1 lower (4.71 lower to 1.5 lower)
CBT-I vs. hypnotics	None	None	None
Patients without underlying diseases			
Insomnia outcome measurements			
CBT-I vs. no treatment			
ISI	455 (6)	⊕⊕⊕⃝moderate	MD 4.88 lower (5.8 lower to 3.95 lower)
CBT-I vs. hypnotics			
ISI	282 (2)	⊕⊕⃝⃝low	MD 2.82 lower (5.22 lower to 0.41 lower)
PQSI	414 (3)	⊕⊕⃝⃝low	MD 0.29 lower (1.21 lower to 0.62 higher)
Depression outcome measurements			
CBT-I vs. no treatment			
BDI	118 (2)	⊕⃝⃝⃝very low	MD 1.19 lower (4.27 lower to 1.89 higher)
CES-D	174 (3)	⊕⊕⃝⃝low	MD 9.58 lower (13.71 lower to 5.45 lower)
CBT-I vs. hypnotics			
HAMD	282 (2)	⊕⊕⃝⃝low	MD 1.27 lower (5.36 lower to 2.82 higher)

## Appendix

  #1 Cognitive Behavioral Therapy [MeSH Terms]
  #2 Behavioral Therapies, Cognitive [Title/Abstract]
  #3 Behavioral Therapy, Cognitive [Title/Abstract]
  #4 Cognitive Behavioral Therapies [Title/Abstract]
  #5 Therapies, Cognitive Behavioral [Title/Abstract]
  #6 Therapy, Cognitive Behavioral [Title/Abstract]
  #7 Therapy, Cognition [Title/Abstract]
  #8 Therapy, Cognitive Behavior [Title/Abstract]
  #9 Cognition Therapy [Title/Abstract]
  #10 Cognition Therapies [Title/Abstract]
  #11 Therapies, Cognition [Title/Abstract]
  #12 Cognitive Psychotherapy [Title/Abstract]
  #13 Cognitive Psychotherapies [Title/Abstract]
  #14 Psychotherapies, Cognitive [Title/Abstract]
  #15 Psychotherapy, Cognitive [Title/Abstract]
  #16 Therapy, Cognitive [Title/Abstract]
  #17 Cognitive Therapies [Title/Abstract]
  #18 Therapies, Cognitive [Title/Abstract]
  #19 Cognitive Therapy [Title/Abstract]
  #20 Cognitive Behavior Therapy [Title/Abstract]
  #21 Behavior Therapies, Cognitive [Title/Abstract]
  #22 Cognitive Behavior Therapies [Title/Abstract]
  #23 Therapies, Cognitive Behavior [Title/Abstract]
  #24 Behavior Therapy, Cognitive [Title/Abstract]
  #25#1OR#2OR#3OR#4OR#5OR#6OR#7OR#8OR#9OR#10OR#11OR#12OR#13OR #14OR#15OR#16OR#17OR#18OR#19OR#20OR#21OR#22OR#23OR#24
  #26 Depression[MeSH Terms]
  #27 Depressions [Title/Abstract]
  #28 Depressive Symptoms [Title/Abstract]
  #29 Depressive Symptom [Title/Abstract]
  #30 Symptom, Depressive [Title/Abstract]
  #31 Symptoms, Depressive [Title/Abstract]
  #32 Emotional Depression [Title/Abstract]
  #33 Depression, Emotional [Title/Abstract]
  #34 Emotional Depressions [Title/Abstract]
  #35 Depressions, Emotional [Title/Abstract]
  #36#26OR#27OR#28OR#29OR#30OR#31OR#32OR#33OR#34OR#35
  #37 sleep initiation and maintenance disorders [MeSH Terms]
  #38 Disorders of Initiating and Maintaining Sleep [Title/Abstract]
  #39 DIMS (Disorders of Initiating and Maintaining Sleep) [Title/Abstract]
  #40 Early Awakening [Title/Abstract]
  #41 Awakening, Early [Title/Abstract]
  #42 Nonorganic Insomnia [Title/Abstract]
  #43 Insomnia, Nonorganic [Title/Abstract]
  #44 Primary Insomnia [Title/Abstract]
  #45 Insomnia, Primary [Title/Abstract]
  #46 Transient Insomnia [Title/Abstract]
  #47 Insomnia, Transient [Title/Abstract]
  #48 Rebound Insomnia [Title/Abstract]
  #49 Insomnia, Rebound [Title/Abstract]
  #50 Secondary Insomnia [Title/Abstract]
  #51 Insomnia, Secondary [Title/Abstract]
  #52 Sleep Initiation Dysfunction [Title/Abstract]
  #53 Dysfunction, Sleep Initiation [Title/Abstract]
  #54 Dysfunctions, Sleep Initiation [Title/Abstract]
  #55 Sleep Initiation Dysfunctions [Title/Abstract]
  #56 Sleeplessness [Title/Abstract]
  #57 Insomnia Disorder [Title/Abstract]
  #58 Insomnia Disorders [Title/Abstract]
  #59 Insomnia [Title/Abstract]
  #60 Insomnias [Title/Abstract]
  #61 Chronic Insomnia [Title/Abstract]
  #62 Insomnia, Chronic [Title/Abstract]
  #63 Psychophysiological Insomnia [Title/Abstract]
  #64 Insomnia, Psychophysiological [Title/Abstract]
  #65#37OR#38OR#39OR#40OR#41OR#42OR#43OR#44OR#45OR#46OR#47OR#4 8OR#49OR#50OR#51OR#52OR#53OR#54OR#55OR#56OR#57OR#58OR#59OR# 60OR#61OR#62OR#63OR#64
  #66#25AND#36AND#65
